# Building capacity in translational research ethics among early-stage investigators: A pilot course on the ethics of the clinician-researcher dual-role

**DOI:** 10.1017/cts.2026.10770

**Published:** 2026-06-04

**Authors:** Laura Stamm, Maya Daniello, Ahona Shirin, Patricia Luck, Mical Raz, Nicholas R. Mercado, Scott McIntosh, Lainie Friedman Ross, Jonathan Herington

**Affiliations:** 1 Department of Medicine, University of Rochester, Rochester, NY, USA; 2 Department of Health Humanities and Bioethics, University of Rochester, Rochester, NY, USA; 3 Department of History, University of Rochester, Rochester, NY, USA; 4 Department of Environmental Medicine and Public Health Sciences, University of Rochester, Rochester, NY, USA

**Keywords:** Clinical researcher, moral decision-making, ethical sensitivity, moral reasoning, responsible conduct of research

## Abstract

**Purpose:**

Translational researchers often occupy dual roles as clinicians and investigators, creating ethical tensions that are inadequately addressed by traditional training in research ethics or clinical ethics. This study aimed to design, pilot, and evaluate a short course to improve early-stage translational researchers’ self-efficacy with respect to navigating conflicts between their roles as researchers and clinicians or community advocates.

**Methods:**

An inter-disciplinary team at University of Rochester developed an 8-module course aimed at improving self-efficacy in moral decision-making. Using Rest’s Four Component Model, the eight course sessions focused on moral sensitivity, reasoning, motivation, and action with respect to dual-role ethical issues such as expanded trial access, ancillary obligations, and return of results. Thirteen early-stage translational researchers participated in a pilot course delivered in person from March through May 2024. Evaluation included pre-and post-course surveys and focus groups.

**Results:**

Participants demonstrated significant improvements in self-efficacy for moral sensitivity (mean increase .79, p < .001), moral reasoning (mean increase .80, p = .001), and ability to take moral action (mean increase .67, p = .002). Participants’ attitudes toward translational research were generally stable. Focus groups revealed that participants highly valued the course, but desired greater curricular cohesion and more guidance on implementation strategies.

**Conclusions:**

This pilot course successfully enhanced early-career translational researchers’ self-efficacy for ethical decision-making in dual-role contexts. The findings suggest that ethics education tailored to translational researchers’ unique challenges can meaningfully improve their capacity to navigate complex ethical tensions.

## Introduction

Translational research involves bringing insights from basic and clinical research to fruition as new interventions, therapies, policies, and practices. The research pathway often involves teams of researchers, including clinicians, basic scientists, data scientists, policy experts, and clinical trial coordinators who work at the boundary of research and clinical care [1]. Often, the principal investigators in these teams work in multiple roles: as both clinician, researcher, and policy advocate [2]. Navigating these multiple roles involves balancing competing goals, which may generate conflicts between duties [3]. For instance, clinicians may attempt to enroll their own patients into a clinical trial. Likewise, researchers working to evaluate public health interventions may act as advocates for policy changes. Researchers working in partnership with community members must also meet their obligations to research funders. As others have noted, applying standard research or clinical ethics frameworks to these problems risks missing important ethical considerations where these dual roles intersect [4–6].

Translational researchers must therefore think carefully about how to balance their responsibilities as researchers with their responsibilities to care for patients and community members. Awareness of the differing obligations and expectations of researchers and clinicians can lead to decision paralysis and moral distress [7]. Understanding how to integrate these two roles, while being faithful to their differing responsibilities, requires translational researchers to pay attention to these tensions, employ tools to address them, and be prepared to partner with patients and research participants in shared decision-making. While researchers receive mandatory training in responsible conduct of human subjects research, and health-practitioners receive training in clinical ethics, rarely do translational researchers receive training on how to navigate their moral duties when these roles intersect.

Previous research has shown that the most effective ethics education occurs in person, with extensive discussion of problems immediately relevant to the learners’ professional practice [8–10]. Additionally, ethics education is more effective when it involves developing learners’ capacity as ethical agents, rather than merely providing knowledge of ethical theory [11]. One prominent theory of ethical decision-making – Rest’s Four Component Model (FCM) – identifies ethical sensitivity, reasoning, motivation, and action as the core constituents of ethical behavior [12–14]. In the context of translational research, “Moral Sensitivity” can be defined as the capacity to identify ethical questions in translational research, making distinctions between different goals and values, and recognizing tradeoffs between the role of clinician and role of researcher. “Moral Reasoning” concerns the capacity to reflect on ethical dilemmas in translational research, including deciding how to prioritize different goals, stakeholders, and roles. “Moral Motivation” captures the propensity of individuals to feel professionally responsible for resolving ethical transgressions in translational research. “Moral Action” refers to the capacity to identify barriers to correct action, and strategies to overcome those barriers. Within the FCM, these constructs are considered jointly necessary for agents to engage in ethical behavior and have formed the foundation of educational interventions designed to improve professional ethics and the responsible conduct of research [ 10,12,15,16].

In this study, we sought to design, pilot, and evaluate a short course to improve translational researchers’ capacity to navigate the intersection of research and clinical ethics. This course explicitly focused on developing junior researchers’ self-efficacy with respect to moral decision-making about conflicts between their role as researchers and their role as clinicians or community advocates.

## Methods

This study was part of a broader research project of the tensions between the role responsibilities of translational researchers. Other components of the broader research included a scoping review and interviews with translational researchers that sought to identify ethical tensions and complementarities between their role as a researcher and their role as a clinician or community advocate. This study was determined to be exempt under the Common Rule by the University of Rochester Human Subjects Research Review Board.

### Course development

The course was collectively developed by a team of six course instructors at the University of Rochester from diverse backgrounds (public health, medicine, bioethics, history, and health humanities), led by the PI/lead instructor. The team met biweekly over the course of 12 weeks to develop a shared understanding of the course goals and identify individual modules. The PI distributed two critical texts prior to the first meeting to shape the team’s discussion and conceptualization of the course [2, 5]. Course instructors then decided how to use their individual expertise to achieve the course aims of improving the FCM constructs with respect to conflicts between the role of researcher and clinician. The initial meetings were a space for team members to brainstorm ideas and give one another feedback. Through this collaborative process, the team identified eight core topics and learning objectives (Table 1).

**Table 1.** Course session topics and learning objectives

Module	Objectives
1. Introduction to Role Conflicts in Translational Research	Describe the philosophical distinctions between duties, norms, and values. Identify the role(s) from which different duties are derived. Describe the core goals and norms of clinical practice and research.
2. Dual-Role Consent	Examine the ethical issues raised by permitting clinicians to enroll their own patients. Describe how the clinician–patient relationship creates potential for bias as well as potential for trust when one has the option of enrolling one’s own patients into research. Discuss the informed consent challenges raised in rare diseases.
3. Expanded Access and Right-to-Try	Describe the primary areas of tension for the physician researcher with expanded access. Determine the ethical risks and benefits of expanded access that impacts patients, researchers, and society. Apply knowledge of expanded-access tensions to an individual patient experience through close reading of a memoir.
4. Pragmatic Clinical Trials and Ancillary Care Obligations	Examine the ethical and regulatory issues raised by pragmatic clinical trials. Discuss whether and when pragmatic trials pose minimal risk/more than minimal risk. Discuss when and to what degree investigators have ancillary care obligations.
5. Secondary Research and Learning Health Systems	Apply the Common Rule’s treatment of secondary research to different cases. Critically evaluate the distinction between research, quality improvement, and treatment. Describe the ethical risks associated with secondary research and apply strategies for navigating ethical risks.
6. Reporting of Research Results	Discuss the challenges translational researchers could face with returning research results to participants. Evaluate the ethical challenges posed by policies and guidelines related to returning primary, secondary, and incidental findings to research participants. Apply the design thinking approach to ethical challenges related to returning research results to participants.
7. Policy and Advocacy	Examine the ethical challenges researchers face when engaging in policy recommendations Describe how research findings might be translated into a specific policy. Identify the ethical issues raised by experts providing (paid) testimony or recommendations deriving from their expertise.
8. Community-Based Participatory Research (CBPR)	Describe the foundational principles of CBPR with regard to investigator and community roles. Identify how CBPR differs from other forms of translational research. Apply strategies for determining participant risk/benefit and responsibility for ethical research practices, to representative cases.

In the following weeks, instructors began to select course readings and develop structured lesson plans to share with the teaching team. Instructors presented these plans during meetings and revised their lesson plan based on feedback. This iterative process ensured each module built upon previous sessions while maintaining consistency in pedagogical approach. The team incorporated diverse teaching methodologies, including Socratic discussion, case studies, close readings of relevant texts, and group problem-solving activities.

Two beliefs guided this course development approach. First, prior work on ethics education has emphasized the importance of creating a safe and collaborative learning environment where participants could explore ethically challenging scenarios [8–10]. The course was thus structured to provide multiple perspectives on each topic, acknowledging that navigating dual-role conflicts often involves complex ethical considerations without clear-cut answers. This approach aligned with the FCM’s emphasis on developing ethical sensitivity, reasoning, motivation, and action capabilities. Second, to ensure the course content was relevant to translational researchers’ experiences, the team developed realistic case scenarios that illustrated common ethical dilemmas encountered when balancing clinical and research responsibilities. These cases were refined through multiple rounds of feedback from the teaching team, with particular focus on creating scenarios that would stimulate discussion and highlight tensions between researcher and clinician roles. Additionally, the team identified pre-reading materials that provided necessary background information while prompting participants to consider how the ethical issues might manifest in their own work.

The PI crafted the final syllabus based on these lesson plans and selected readings, organizing the course into a coherent progression that would build participants’ ethical capacities. Table 1 provides an overview of each module’s learning objectives. Most course sessions involved a collaborative discussion of multiple case studies, informed by a theoretical pre-reading, which focused on asking participants (i) what ethical risks existed in the case (sensitivity), (ii) how might we apply the theoretical tools from the reading (reasoning), and (iii) and what barriers or facilitators existed with respect to deploying those tools (action). Additionally, one course session involved a design thinking exercise to generate a policy on incidental finding disclosure, another incorporated a close reading of a patient narrative of their experience in an “expanded access” oncology trial, and a third involved the development of mock policy briefs. A full description of each module is provided in Appendix 1.

### Recruitment

Participants were recruited through an emailed open call. This call was distributed through the heads of the post-graduate clinical and research training programs at University of Rochester Medical Center (URMC). This included the KL2 program, the Rochester Early-Stage Investigator Network, the MD-PhD program, and the residency and fellowship program directors of the full range of clinical specialties, including neurology, surgery, palliative care, medicine, and pediatrics. Additional targeted outreach to potential participants was performed through department heads, research directors, and the diversity, equity and inclusion office. Because URMC is primarily a clinical institution, early career investigators occupying dual roles typically have substantial clinical responsibilities. Recruitment thus focused on KL2 scholars, K01 awardees, fellows, and other researchers with protected time to dedicate to research ethics training.

Potential participants were asked to complete a pre-screening survey (administered through REDCap) to establish their eligibility and provide contact details [17]. Seventeen potential participants identified themselves. Because participants were busy trainees and early-stage investigators, the potential participants were given the opportunity to express preferences for the timing and dates of the eight two-hour sessions. After scheduling, four potential participants dropped out. The remaining 13 participants confirmed their participation via email.

Course participants were informed they would also be research participants, and they would be required to complete two surveys and participate in a 90-minute focus group. Participation was incentivized with a $1000 honorarium for full completion (pro-rated incentives were offered for early withdrawal).

### Course evaluation

The evaluation team iteratively designed and pretested the course evaluation pre-and post-survey. Each iteration incorporated feedback from the other course facilitators and survey experts. A pre-survey was administered prior to the start of the course to familiarize learners with course objectives and provide baseline data. A post-survey was administered after the course to evaluate their learning and effectiveness of course modules.

In both surveys participants were asked to complete three main sections. First, they were asked to rate their degree of agreement (5-point Likert, “Strongly Disagree” to “Strongly Agree”) with six statements about the social value and regulation of translational research (Table 2). Second, they were asked to identify the degree to which (10-point Likert, from “Not at all” to “Extremely” challenging) nine different translational study designs were ethically challenging (Table 3). Lastly, they were asked to rate their degree of agreement (5-point Likert, “Strongly Disagree” to “Strongly Agree”) with a set of items that collectively sought to capture their self-efficacy with respect to four FCM constructs in the context of translational research: (i) moral sensitivity, (ii) moral reasoning, (iii) moral motivation, and (iv) moral action (See Supp Tables 1, 2 and 3).

**Table 2.** Attitudes toward translational research

Item question	Before the course *mean* (CI)	After the course *mean* (CI)	*p* Value*
Clinical and translational research projects generally benefit their research participants.	3.54 ±.51	2.58 ±.41	<.01
Clinical and translational research projects generally produce valuable scientific knowledge.	4.15 ±.40	3.75 ±.38	.10
Clinical and translational research projects are mostly motivated by investigator self-interest (i.e., publications, promotion, patents, and drug approval).	3.08 ±.60	3.08 ±.66	.79
The regulations regarding clinical and translational research projects are clear.	3.08 ±.50	2.92 ±.66	.78
Principal investigators are ultimately responsible for the ethical conduct of a research project.	4.62 ±.38	4.33 ±.60	.10
The Institutional Review Board is a barrier to the conduct of clinical and translational research.	2.23 ±.48	2.50 ±.41	.27

* *p*-Values for difference between the population mean of each item before or after the course were calculated as a two-sided paired *T*-test.

**Table 3.** Perceived degree of ethical challenge

How ethically challenging are… *(1 = not at all, 10 = extremely)*	Before the course *mean* (CI)	After the course *mean* (CI)	*p*-Value*
Phase 1 clinical trials	7.31 ± 1.24	6.83 ± 1.37	.29
Phase 3 clinical trials	6.62 ±.99	6.36 ± 1.00	.44
Pragmatic trials in a hospital setting	6.46 ± 1.25	6.18 ± 1.18	.09
Pragmatic trials in a primary care setting	5.38 ± 1.60	5.82 ± 1.38	.82
Qualitative studies with community groups	5.31 ± 1.24	5.45 ± 1.42	.66
Patient reported outcome studies	4.69 ± 1.10	4.91 ±.70	.59
Secondary research w/de-identified genomic data	4.31 ± 1.17	4.82 ±.72	.77
Secondary research with deidentified EHR data	2.54 ± 1.18	4.67 ± 1.31	.01
Anonymous survey research	2.77 ± 1.30	2.92 ± 1.14	>.99

*p-Values for difference between the population mean of each item before or after the course were calculated as a two-sided paired *T*-test.

These items capture “self-efficacy” (i.e. the individual’s own perception of their capacity) and were not designed to assess the *form* or *quality* of moral reasoning (e.g., Rest’s Schema’s), sensitivity, or motivation. Rather, they capture participants’ subjective confidence in their ethical capacities within the translational research context. We chose not to employ existing instruments that directly measure components of the FCM (such as the Defining Issues Test), because these purport to assess general moral decision-making rather than moral decision-making specific to clinician-researchers. Instead, we sought to capture self-efficacy in sensitivity, reasoning, etc., in the specific context of dual-role ethics. While improvements in self-efficacy are conceptually distinct from changes in underlying moral reasoning ability, there is good evidence that self-efficacy is a strong predictor of underlying capacity and behavior change [18–20]. Self-efficacy has been shown to be a consistent predictor of subsequent health-related outcomes, including physical activity, eating habits, smoking, and drinking alcohol [21].

In the post survey, participants were asked to evaluate their self-efficacy for each item both “before” and “after” the course: in other words, they were asked to look-back with the assumption that they could perhaps now more accurately rate their self-efficacy. The scores for items in the Pre-survey compared to the parallel ‘Before’ (look-back) items in the Post Survey were substantially equivalent. In the analysis, we compared pre-course responses to the post-course “After” responses.

Prior to analysis, all survey responses were deidentified. Frequencies were computed for all individual items (categorical, Likert-type), and participants’ scores for each FCM construct were computed by averaging the ordinal rank of their response to each item within a construct (i.e., where “Strongly Disagree” =1, and “Strongly Agree” = 5). Following the practice of treating well-behaved Likert-scale responses as interval data, descriptive statistics for the study population (i.e. mean, and 95% confidence intervals) were computed for each attitudinal item, ethical challenge item, FCM item, and FCM construct [22]. A two-sided paired *T*-test was used to test for statistically significant changes between the scores prior to the course and after the course.

The evaluation team also conducted qualitative focus group studies to glean more nuanced feedback from course learners that could not be captured in the survey. Focus group interview guides were developed and pre-tested by the investigators to ensure questions adhered to qualitative research methods (exploratory, open-ended, not double-barreled, no redundancy, optional prompts). The interview included four sections: Overall impression, course content, course format, and barriers and facilitators to learning (Appendix 2). Questions asked participants to reflect on how this course compared to other research ethics education they received; which instructors, topics, and/or formats they found most valuable; and the balance between philosophical and practical considerations. They were also asked whether they would recommend the course to a colleague, along with what type of clinician and/or researcher would benefit most from this type of ethics education. Two semi-structured focus groups were held in person with each of the course cohorts, with some learners needing to attend the other session to accommodate their schedule. The focus group was facilitated by one of the team’s research assistants who was familiar with the course content but had not previously interacted with the learners. Another research team member took notes for each focus group.

Recordings were transcribed verbatim using automated transcription software and reviewed by a research assistant. Quotations included in this manuscript were lightly edited for clarity, removing repeated filler words (e.g. “um,” “like”). ATLAS.ti (Ver #24.1.1) qualitative coding software was used to implement an inductive coding strategy based on a Grounded Theory approach and consistent with our previous work [23–25]. Five experienced coders worked collaboratively and iteratively to reach consensus in the identification of themes across the focus group transcripts.

## Results

Participants (*n* = 13) were recruited into the study, all of whom completed both the survey and focus group. Nine of the 13 respondents (69%) self-reported as women. Two respondents identified as Asian or Pacific Islander (15%); two were Black (15%); seven were White (54%); three identified as a race other than the OMB list, and one declined to answer. Two respondents self-reported as Hispanic (15%). Average age of participants was 34 (SD = 5.0), and average years of experience in the field (as measured from terminal degree) was 5.6 years (SD = 4.3). The cohort included four KL2/K01 scholars, four medical fellows, three postdoctoral scholars, and two unfunded early-career clinical faculty with emerging research programs. Specialties represented included neurology, surgery, pediatrics, nursing, and public health. There were seven participants with MDs, five with PhDs, and a physician assistant with a BA/MS terminal degree.

### Attitudes toward translational research

Participants’ reported attitudes toward translational science were generally stable (Table 2). There was no significant change in participant confidence in the overall value, impartiality, and regulation of translational research projects. The only significant change was toward whether clinical and translational research benefits the *research participants*, with almost half of respondents indicating agreement before the course, and none of the participants agreeing or strongly agreeing after the course. We suspect that this is a result of discussions about “therapeutic misconception” during several sessions, which may have induced participants to consider perceived benefits to participants as an ethical risk to be minimized as part of clinical research.

Prior to the course, participants rated Phase 1 and pragmatic trials as the two most ethically challenging research activities, while secondary and anonymous data research were considered the least challenging. There was no significant change in these attitudes after the course except for secondary research with de-identified EHR data, where participants increased their rating of its difficulty by an average of 2.00 ± 1.35 (Table 3). We suspect that this was due to the course session on secondary research ethics, which emphasized the possibility of re-identification and “group risks” as a result of research using de-identified health data [26]. Despite this change, participants still rated Phase 1 Clinical Trials as the most ethically challenging overall.

### Moral decision-Making self-efficacy

**Figure 1.**
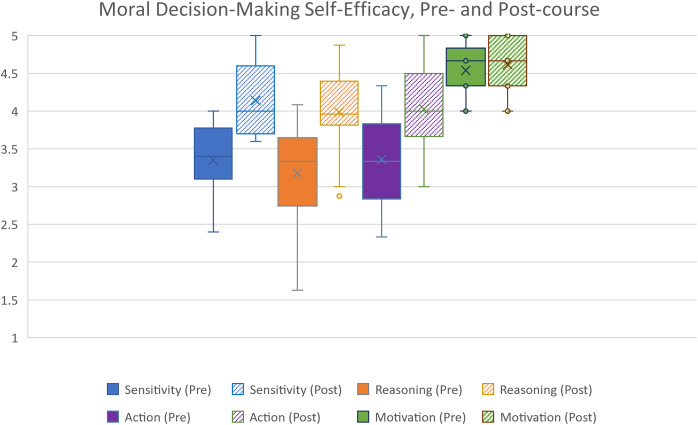
Moral decision-making self-efficacy, pre-and post-course.

Participants’ self-efficacy with respect to moral sensitivity, moral reasoning, and moral action all experienced a positive shift from pre-to post-course answers (Figure 1). Participants’ moral sensitivity self-efficacy showed the largest overall change of the three categories (Table 4). Results showed an increase in participant confidence in their ability to differentiate between patient and research participant roles and clinician and researcher roles, and to identify conflicts of interest, issues of trust, and power dynamics involved in conducting research. The biggest change can be seen in participants’ perceived ability to distinguish between their distinct roles as clinicians and as researchers with an average change of 1.54 (.51) (Table S1).

**Table 4.** Self-efficacy for moral decision-making constructs

Construct	Before the course *mean* (CI)	After the course *mean* (CI)	*p* Value*
Moral sensitivity	3.35 ±.27	4.14 ±.28	<.001
Moral reasoning	3.18 ±.41	3.98 ±.33	.001
Moral motivation	4.54 ±.20	4.62 ±.21	.285
Moral action	3.36 ±.35	4.03 ±.31	.002

*p-Values for difference between the population mean of each construct before or after the course were calculated as a two-sided paired *T* test.

Participants reported a significant increase in their overall self-efficacy with respect to moral reasoning (Table 4). The biggest change occurred in their confidence to make appropriate ethical judgments about clinical and translational research and their confidence to act ethically when a person is both a patient and research participant (Table S2). When participants were asked to think about specific parts of the research process, the results were more variable. Participants noted increased confidence with moral judgements regarding protecting participants from adverse outcomes and identifying appropriate study topics. There was, however, little change in their ability to report back incidental findings, report results to the scientific community, and report-back results to patients/community members.

Similarly, participants reported an increase in their ability to view responsible conduct of translational research as within their control (moral action). At the item level, there was no change in participants’ feeling that navigating ethical dilemmas is within their control, but significant changes in their perceived ability to overcome barriers to responsible conduct and access resources to help them navigate ethical challenges (Table S3). Finally, there was no significant change in participants’ sense of their responsibility to act ethically (moral motivation), at either the construct or individual item level (Table S3).

### Qualitative experience with course

Overall, participants felt that the course exceeded expectations, especially in comparison with an existing, mandatory ethical conduct of research course (known as “IND 501”). They reported that this course catered to the needs of translational researchers, whereas the required research ethics course responds primarily to issues faced by bench scientists. Participants additionally shared that one of the most valuable aspects of the course was the opportunity to learn from peer translational researchers working across different departments and areas of research.

…this seemed a lot more useful for our kind of investigator mixed methods, [IND 501] was almost more for like bench science research” (Participant A).

“I think in many instances for me, I don’t know that I would have interfaced with other participants. We don’t overlap scientifically. We don’t overlap domain wise. We would have never met… you wouldn’t have had interacted with people. And it’s kind of hard to get that at this stage… I’m still a new faculty member as well, [so] it’s nice to meet other people (Participant M).

The participants most valued the sessions on the tension between the clinician-investigator roles and the ethics of community-based participatory research (CBPR). Despite these topics being outside some of the participants’ own areas of research, they found these topics translatable to their experiences and commented that they provided a new lens through which to view translational research.

… CBPR—it’s not new, but it’s new-ish, and compared to perhaps dual consent, I feel like there’s just so much there. I did find myself after that session in particular wanting more discussion, more readings, more understanding (Participant J).

… I found it fascinating, the tensions between being a clinician and a researcher. So, even though I don’t do a lot of clinical work, it’s certainly brought to mind the problems that could exist if you’re doing both and recruiting your own patients. What do you distinctly, you know? Which part of it then takes priority for you? (Participant L).

Participants expressed ambivalence toward the sessions that explored philosophical and theoretical ethics, preferring instead more discussion of the practical implementation of those ethics. The ambiguous nature of the theoretical discussion left participants feeling confused on how to apply what they had discussed, and participants agreed that they would have benefitted from a session that focused on different ways of implementing ethics in their own work. They suggested including case studies personalized to the research questions of course participants for a future iteration of the course to allow for this type of immediate application.

I feel like with a lot of the things I’m still grappling with… what to advocate for change, what of the structures that we have to support the ethical conduct of research really are no longer appropriate, you know, and I guess I came in sort of wanting that but like, what should we do next? How could we apply this to our work or our advocacy…? (Participant M).

Participants least preferred sessions in which the goals of the session or underlying message were perceived to be unclear. They suggested structured readings or summaries alongside the discussion to help facilitate comprehension of the more challenging concepts.

…there was sort of this very open-ended discussion where I feel like none of us quite knew what we were supposed to be getting out of it (Participant K).

Participants most preferred the formats that allowed for collaborative learning and discussion, such as the Socratic method, “chalk and talk,” and open discussion with time for asking questions and engaging with each other. These were found to be the most engaging formats and most valuable for integrative understanding of the complex topics.

The classes were so rich and engaging with this sort of conventional format. And I always felt like there was never any dead air, you know, like we always had so many different ideas percolating and things to talk about (Participant K).

The main criticism of the course was the perceived lack of cohesion among topics and presenters throughout the curriculum. The participants shared that a more consistent curriculum, with shared presentation styles, would have made it easier to move within discussions and switch topics week-to-week. They stressed that the different session formats (didactic, open discussion, group work, close reading, etc.) presented the greatest challenge to their learning.

And presentation materials vary widely. Readings vary widely… It didn’t feel like there was a lot of consensus. And maybe that’s part of it is that you didn’t want that but was very apparent that different people were teaching different weeks, I guess (Participant M).

The largest expressed barrier to course participation expressed was the time burden. Participants had to fit the required coursework into their normal work week, sometimes without the support of their division chief and/or institution. Participants felt that this was a large barrier to their success in the course.

Unfortunately, the reality of it is the biggest barrier [is] those that should do the course are those that unfortunately can’t show up. And so, I think that’s really hard because it self-selects a very specific group of people that have the flexibility and privilege to be able to just show up and talk about ethics for two and a half hours (Participant J).

## Discussion

This pilot course successfully enhanced early-career translational researchers’ self-efficacy for ethical decision-making in dual-role contexts in which investigators must manage competing moral obligations [ 3,27,28]. As other scholars have noted, dual-role investigators face unique ethical challenges, such as consenting their patients for both care and research [ 29,30]. While others have called for increased education, this course is the first to pilot and evaluate the effectiveness of ethics training to help investigators navigate competing duties [ 31,32]. The findings suggest that ethics education tailored to translational researchers’ dual-role can meaningfully improve their capacity to navigate complex ethical tensions.

Course participants often expressed eagerness to engage with translational research ethics but described how prior research ethics courses did not adequately address the unique challenges faced by translational researchers [33]. As the framing of the course emphasizes, translational researchers often face dual-role conflicts not experienced by basic scientists. Working in partnership with community members and patients, for example, creates new ethical challenges for translational researchers and shifts the way they might conceive of research risk and benefit [34]. Survey results demonstrated that course participants shifted their belief that clinical and translational research projects generally benefit their research participants. This shift may be attributed to the course ending with sessions on public policy and advocacy and CBPR, both of which emphasized translational researchers’ obligations to the public [35]. Participants, then, likely left with the belief that research should have benefits for the public and community beyond research subjects receiving a particular health intervention [36].

Similarly, the course session on secondary research and learning health systems likely led to the shift in participants’ perceptions about the degree of ethical challenge posed by secondary research with deidentified EHR data. While participants still deemed Phase 1 Clinical Trials the most ethically challenging, they found secondary research with deidentified EHR data significantly more ethically challenging after the course. Secondary research is generally deemed exempt from Institutional Review Board (IRB) human subjects review because it is considered low risk; however, the course session demonstrated that even when data are deidentified, potential harm can be done to individuals and communities [37]. The rapid increase of artificial intelligence (AI) in health care and implementation of AI to achieve a learning health system raises additional ethical concerns related to health disparities, stigmatization, and social value [38]. Translational researchers thus need training in these emerging ethical issues to conduct research that does not harm the patients and communities they serve.

As course participants began to think about translational research ethics and their dual roles with increased nuance, they expressed concerns about interacting with IRBs who may or may not be familiar with specifics of their research and particular ethical concerns. Those conducting community-engaged research felt a lack of understanding and, sometimes, lack of compatibility between their research aims and the IRB strictures [ 39,40]. To address these perceived challenges, participants suggested future iterations of the course include interaction with IRB members. Their desire for interaction with IRBs could also be met through research ethics consultation, a more collaborative, conversational process than the traditional IRB review [41]. Participants also expressed their desire for more senior researchers and administrators to receive education on translational research ethics so that mentorship and institutional policies more appropriately respond to their needs.

### Limitations

Several limitations warrant consideration. First, our survey instruments measured participants’ self-reported confidence in their ethical capacities rather than their actual moral sensitivity, reasoning, or behavior. While self-efficacy is an important predictor of professional behavior, it may not correspond directly to improved ethical decision-making. Future studies should incorporate measurement of self-efficacy alongside behavioral assessments or case-based evaluations that aim to uncover underlying FCM capacities. Second, our sample size of 13 participants limits the generalizability and statistical stability of our findings. Small samples in educational research are susceptible to individual variation, and our results should be interpreted as preliminary. Larger-scale replication of the course, with diverse cohorts across multiple institutions, would strengthen confidence in these findings. Third, the FCM framework, while useful for structuring curriculum, does not fully account for the role of emotion, uncertainty, power dynamics, or institutional factors in ethical decision-making. Alternative approaches, such as reflecting on the virtues of a clinician-investigator, adopting decision-making strategies, or improving institutional cultures, may be more effective for certain types of dual-role conflicts than individual-level moral reasoning training [16,42]. Finally, the self-selected nature of our participants may introduce bias; those who enrolled likely had pre-existing interest in research ethics, potentially limiting applicability to less motivated populations.

### Future directions

These limitations notwithstanding, this pilot course demonstrates one way to equip early career researchers with the confidence to responsibly conduct human subjects research when they occupy dual roles as both researchers and clinicians or community advocates. The pilot course was conducted with a second, co-occurring project aim, in which the research team conducted qualitative interviews with clinical and translational researchers on the ethical challenges they face when occupying dual roles. Future iterations of the course module will incorporate insights gained from interview analysis, along with refined evaluation methods. Subsequent iterations will also seek to recruit different types of researchers working within different contexts, including MD-PhD students. This type of training may better prepare future clinician-scientists to navigate the complex ethical terrain of their dual roles as both clinicians and researchers.

## Conclusion

As the boundaries between clinical care and research continue to blur, and community-engaged methods that prioritize benefit-sharing proliferate, the challenges of balancing different moral roles will become more pronounced. This study aimed to design, pilot, and evaluate a course to improve translational researchers’ ability to ethically navigate their dual roles as researchers and clinicians or community advocates. The findings demonstrate that such education improves early-stage investigators’ ethical self-efficacy. While improved self-efficacy does not solve the underlying ethical dilemmas, we hope that greater confidence in their moral sensitivity, reasoning, motivation and action will help clinician-researchers appropriately identify dual-role ethical dilemmas, discuss them openly with peers, and advocate for individual and structural mechanisms for promoting responsible conduct. This course is a first step, but we believe it is a valuable one.

## Supporting information

10.1017/cts.2026.10770.sm001 Stamm et al. supplementary material Stamm et al. supplementary material

## Data Availability

No data was received from outside sources.

## Supplementary material

The supplementary material for this article can be found at https://doi.org/10.1017/cts.2026.10770.
