# Supplementary material for: Building capacity in translational research ethics among early-stage investigators: A pilot course on the ethics of the clinician-researcher dual-role
Source: J Clin Transl Sci. 2026 Jun 4;10(1):e111. doi: 10.1017/cts.2026.10770 (PMC13430488; doi:10.1017/cts.2026.10770)
Supplement: Stamm et al. supplementary material [file S2059866126107705sup001.docx]

Building capacity in translational research ethics: a pilot course on the ethics of the clinician-researcher dual-role

Stamm, Laura; Daniello, Maya; Shirin, Ahona; Luck, Patricia; Raz, Mical; Mercado, Nicholas; McIntosh, Scott; Ross, Lainie; Herington, Jonathan

# Supplementary materials

## Appendix 1: Description of Course Materials

Module one, "Introduction to Role Conflicts in Translational Research," provided the foundation for exploring the ethical tensions faced by researchers who occupy multiple roles simultaneously. Through a structured icebreaker activity and collaborative group work, participants identified their professional identities, the goals associated with those identities, and their principal ethical concerns. The module introduced participants to the concept of "moral spheres" as described by Doernberg and Truog,^1^ examining how different roles (clinical care, research, and public/community health) each have distinct goals, primary stakeholders, and governing ethical norms. Through case-based discussions exploring scenarios like COVID-19 hospitalization modeling and clinical trial enrollment, participants applied frameworks for navigating role conflicts, including the traditional "clinician as fiduciary" approach, the "equality of roles" perspective, and a "duty-based framework" that distinguishes between intra-personal and inter-personal conflicts. This introductory module established essential terminology and conceptual frameworks that would be developed throughout the course.

Module two, “Dual-Role Consent,” explored the ethical issues raised by the enrollment of one’s own patients into a study one is also conducting.^2^ The module began with an overview of the challenges raised by dual-role consent, as well as the arguments supporting role synergy: the belief that the combination of two roles is more effective than the individual parts alone. The group discussed the conflicts raised by dual role consent process and how they can be minimized.^3^ This discussion explored the challenges faced by the patient-participant (including challenges to voluntariness and how to avoid or minimize the therapeutic misconception) and by the clinician-researcher (including how to maintain equipoise, align research with the clinical fiduciary responsibility to maximize health interest, and present information in a non-biased manner). The concept of a neutral third party to obtain consent was introduced, as well as its feasibility in the rare disease setting.

Module three, “Expanded Access in Translational Research,” focused on how Expanded Access allows patients to be treated with investigational medical interventions when approved options are inadequate and trial participation is unavailable. Unlike the primary goal of research, which aims to advance generalizable knowledge and offers only incidental benefit (if any) to study participants, the primary goal of expanded access is to benefit patients.^4^ Through small group discussions, this module explored the challenges for the clinician-researcher when considering Expanded Access and the tension that arises in caring for a patient while also protecting the integrity of clinical trials. The three areas discussed in class explored the benefit to the patient, benefit to clinical research, and benefit to society. The second half of the module focused on how participants might initiate a conversation on Expanded Access and navigate shared decision making with patients through a close reading of a memoir detailing one author’s illness and treatment experience.^5^

Module four, “The Provision of Clinical Care in Research: From Pragmatic Clinical Trials to Ancillary Care Obligations,” centered the provision of clinical care in the research setting. Clinical care may be provided in the research setting through pragmatic clinical trials where two standard of care treatments are being compared.^6^ Course participants considered the two opposing positions: 1) these trials are safe and pose minimal risk, and that such research can be exempted from institutional review and a rigorous informed consent process; and 2) these trials require traditional informed consent because they include additional risk caused by research participation, and the failure to do so promotes the therapeutic misconception.^7^ A second way in which clinical care may be provided is through ancillary care obligation.^8^ Ancillary care is the provision of clinical care needed by research participants but not necessary to ensure scientific validity, prevent study related harms, or address study-related injuries. Participants explored whether ancillary care is owed, and if so, the limits to this care provision.

Module five, "Secondary Research and Learning Health Systems," explored the ethical considerations of using pre-existing data for new research purposes, with a particular focus on machine learning applications and quality improvement studies within healthcare settings. The session began with a case-based discussion of a "health need predictor" algorithm, guiding participants through the process of identifying potential ethical risks such as identifiability concerns, consent requirements, potential harms to individuals, health inequities, and stigmatization. Through collaborative examination of regulatory frameworks, participants learned to distinguish between quality improvement and research activities, and to categorize data as identifiable, coded, de-identified, or anonymous according to Common Rule and HIPAA standards. The module then introduced Faden's seven fundamental obligations for ethical conduct within learning healthcare systems, prompting critical discussion about the tension between individual patient protections and community benefits.^9^ Throughout the session, participants engaged with questions about when secondary research requires notification or consent, how to balance data representativeness with community trust, and what ethical procedures should guide de-identified data analysis.

Module six, “Reporting of Research Results,” examined the ethical challenges translational researchers might face when disclosing research results to their participants.^10^ The first half of the session included a discussion of returning results to patients and research participants, analyzing guidelines for return of results through a case study on incidental findings and the challenges of disclosure. The second half included a design thinking workshop, a problem-solving activity that engages a human-centered discovery process to foster creativity.^11,12^ In this design thinking workshop, learners were divided into two groups and were tasked to develop an innovative solution to one of the ethical issues identified in class. They were asked to explore why the issue was important to address (empathize), articulate their group’s point of view (define), brainstorm creative ideas for a solution (ideate), and describe a practical structure for their idea (prototype). Near the end of the module, the two groups presented their innovations to the larger class and discussed their creative process (test).

Module seven, “Policy and Advocacy,” was designed to support researchers in thinking through the policy motivations that might underpin their work, and how their work might be translated into evidence-based policies. Numerous fields of research render results that have potential policy implications that could help to improve our population’s health, well-being, and longevity. Yet science is never neutral, and harnessing research findings for policy development has ethical pitfalls. The participants critically considered the interchange between research and policy and took time to carefully consider unintended consequences. The module then turned to a case-based discussion, as participants worked in groups to develop potential policy recommendations, additionally considering whether these policies might have the intended impact or not and downstream consequences they might create. In this session, participants discussed how researchers might think about their research findings and the potential for informing policy debates.^13–15^

Module eight, “Community-Based Participatory Research (CBPR),” ended the course by exploring how engaging community partners in research complicates many of the foundational principles of human subject research ethics.^16,17^ Specifically, this module asked how researchers balance their dual roles as investigators and community collaborators. This dual role raises several tensions for researchers: allowing community partners and participants to shape the research process while maintaining investigator responsibilities, navigating the tension between community partners’ focus on the “CBP” with their focus on the “R”, and meeting ethical commitments to the IRB while prioritizing mutual benefit for community.^18^ After establishing a shared understanding of researchers’ ethical obligations to community, clinical research, and research ethics, the module turned to case studies based in Rochester community research priorities. This final module built upon previous sessions by asking participants to reflect upon how their ethical commitments as a researcher and/or clinician may become re-oriented when research is based in community.

**References**

1. Doernberg S, Truog R. Spheres of Morality: The Ethical Codes of the Medical Profession. *Am J Bioeth*. 2023;0(0):1-15. doi:10.1080/15265161.2022.2160514

2. Morain SR, Joffe S, Largent EA. When Is It Ethical for Physician-Investigators to Seek Consent From Their Own Patients? *Am J Bioeth*. 2019;19(4):11-18. doi:10.1080/15265161.2019.1572811

3. Berman SA, Garbayo L. Revising Our Standards on Dual-Role Physicians: Proceed, but Proceed With Caution. *Am J Bioeth*. 2019;19(4):49-50. doi:10.1080/15265161.2019.1572824

4. Polak TB, Fernandez Lynch H. The Ethics of Expanded Access Research. *JAMA*. 2023;329(13):1057. doi:10.1001/jama.2023.2204

5. Yip-Williams J. *The Unwinding of the Miracle: A Memoir of Life, Death, and Everything That Comes After*. Random House; 2019.

6. Kim SY, Kimmelman J. Practical steps to identifying the research risk of pragmatic trials. *Clin Trials*. 2022;19(2):211-216. doi:10.1177/17407745211063476

7. McKinneyJr RE, Beskow LM, Ford DE, et al. Use of altered informed consent in pragmatic clinical research. *Clin Trials*. 2015;12(5):494-502. doi:10.1177/1740774515597688

8. Belsky L, Richardson HS. Medical researchers’ ancillary clinical care responsibilities. *BMJ*. 2004;328(7454):1494-1496. doi:10.1136/bmj.328.7454.1494

9. Faden RR, Kass NE, Goodman SN, Pronovost P, Tunis S, Beauchamp TL. An ethics framework for a learning health care system: a departure from traditional research ethics and clinical ethics. *Hastings Cent Rep*. 2013;43(s1):S16-S27. doi:10.1002/hast.134

10. Schaefer GO, Savulescu J. The Right to Know: A Revised Standard for Reporting Incidental Findings. *Hastings Cent Rep*. 2018;48(2):22-32. doi:10.1002/hast.836

11. Marcus D, Simone A, Block L. Design thinking in medical ethics education. *J Med Ethics*. 2020;46(4):282-284. doi:10.1136/medethics-2019-105989

12. Brown T. Design thinking. *Harv Bus Rev*. 2008;86(6):84-92, 141.

13. Rubin D. Developing Policy When Evidence Is Lacking. *JAMA Pediatr*. 2016;170(10):929-930. doi:10.1001/jamapediatrics.2016.1945

14. Connelly S, Vanderhoven D, Rutherfoord R, Richardson L, Matthews P. Translating research for policy: the importance of equivalence, function, and loyalty. *Humanit Soc Sci Commun*. 2021;8(1):191. doi:10.1057/s41599-021-00873-z

15. Kilbourne AM, Garrido MM, Brown AF. Translating research into policy and action. *Health Serv Res*. 2022;57(S1):5-8. doi:10.1111/1475-6773.13980

16. Horowitz CR, Robinson M, Seifer S. Community-based participatory research from the margin to the mainstream: are researchers prepared? *Circulation*. 2009;119(19):2633-2642. doi:10.1161/CIRCULATIONAHA.107.729863

17. Tamariz L, Medina H, Taylor J, Carrasquillo O, Kobetz E, Palacio A. Are Research Ethics Committees Prepared for Community-Based Participatory Research? *J Empir Res Hum Res Ethics*. 2015;10(5):488-495. doi:10.1177/1556264615615008

18. Eder MM. Aligning clinical research ethics with community-engaged and participatory research in the United States. *Front Public Health*. 2023;11:1122479. doi:10.3389/fpubh.2023.1122479

## Appendix 2: Focus Group Script

NOTE: *question in italics are possible follow-ups depending on content of answer.*

**OVERALL IMPRESSIONS**

1. What were your overall impressions of the course curriculum?
   1. *How does this course compare to other research ethics education you have received? (e.g. for credit-courses, CPE)*
   2. *Was there anything about the course that was especially unique or distinctive? If so what?*
   3. *Anything other overall impressions?*
2. What aspects of the course did you find most valuable?

**COURSE CONTENT**

*Distribute the syllabus sheet with a summary of each week’s topic and readings.*

1. What topics did you find most valuable?
2. No course can cover everything about research ethics, so tell me about any topics that you wish we had covered.
   1. *Tell me about any topics that were discussed in class that you wish we had spent more time on.*
3. What recommendations would you have for improving the course content?

**COURSE FORMAT**

1. Tell me about any particular class activities or formats that stood out.
   1. *e.g., the didactic learning (e.g. use of powerpoint / handouts to explain key concepts)*
   2. *e.g., the case-driven discussions)*
   3. *e.g., the design-thinking exercise*
   4. *e.g., the deep reading exercise*
   5. *What do you feel could have been done differently with respect to class activities?*
2. How effective were the course instructors?
   1. …*at communicating key concepts?*
   2. *…at facilitating discussions?*
   3. *…at fostering productive dialogue?*
   4. *What do you feel instructors could have done differently?*
3. Tell me about how effectively the course balanced theoretical/philosophical discussions with practical/real-world application.
   1. *What did you think about the level of depth in the philosophical/theoretical discussions?*
   2. *Tell me about any practical strategies that you will use in your work as a clinician-researcher.*
   3. *What do you feel could have been done differently to improve the practicality of the course?*

**Course Future**

1. Would you recommend this course to your colleagues? (Raise hands)
   1. *Why? Why not?*
2. What type of clinician or researcher do you think would benefit most from taking this program?
3. Would you be a facilitator for such a course in the future?
4. Any other changes you would make?
5. Any other thoughts?

**Table S1**: Distribution of Likert Scores by Item for Moral Sensitivity Construct

|  | **Before the Course**  **(n)** | | | | | **After the Course**  **(n)** | | | | |
| --- | --- | --- | --- | --- | --- | --- | --- | --- | --- | --- |
| **Item** | **Strongly Disagree** | **Disagree** | **Neutral** | **Agree** | **Strongly Agree** | **Strongly Disagree** | **Disagree** | **Neutral** | **Agree** | **Strongly Agree** |
| I feel confident in my ability to differentiate whether I am working with an individual as a patient or as a research participant | - | - | 5 | 8 | **-** | - | **-** | 2 | 7 | 4 |
| I feel confident in my ability to identify when I am caring for people and when I am studying them. | - | 1 | 5 | 6 | 1 | - | - | 3 | 7 | 3 |
| I feel confident in my ability to identify instances where there are conflicts between my duties as a researcher and my duties as a clinician. | - | 2 | 4 | 6 | - | - | - | - | 9 | 4 |
| I feel confident in my ability to identify power differentials between participants and researchers. | - | 2 | 3 | 7 | 1 | - | - | **-** | 6 | 7 |
| I feel confident in my ability to identify when research participants have lost trust in me. | - | 8 | 1 | 4 | - | - | 3 | 2 | 4 | 4 |
| **Moral Sensitivity** | **0%** | **20%** | **28%** | **48%** | **3%** | **0%** | **5%** | **11%** | **51%** | **34%** |

**Table S2**: Distribution of Likert Scores by Item for Moral Reasoning Construct

|  | **Before the Course**  **(n)** | | | | | **After the Course**  **(n)** | | | | |
| --- | --- | --- | --- | --- | --- | --- | --- | --- | --- | --- |
| **Item** | **Strongly Disagree** | **Disagree** | **Neutral** | **Agree** | **Strongly Agree** | **Strongly Disagree** | **Disagree** | **Neutral** | **Agree** | **Strongly Agree** |
| I feel confident that I can make appropriate ethical judgments about my clinical and translational research projects. | - | 2 | 6 | 5 | - | - | - | 2 | 7 | 4 |
| I feel confident that I can decide how to act ethically when an individual is both my patient and my research participant. | - | 4 | 4 | 5 | - | - | 1 | 1 | 8 | 3 |
| I feel confident in my judgments about how to: |  |  |  |  |  |  |  |  |  |  |
| ...identify topics of study that benefit my patients / community." | 1 | 1 | 2 | 9 | - | **-** | **-** | 2 | 9 | 2 |
| ...design studies that balance protecting research participants and conducting useful science. | 1 | 2 | 7 | 3 | - | - | 1 | 3 | 7 | 2 |
| ...conduct informed consent conversations with research participants. | 2 | - | 4 | 5 | 2 | - | **-** | 2 | 7 | 4 |
| ...report back incidental findings to research participants | 1 | 5 | 3 | 4 | - | - | 1 | 4 | 8 | - |
| ...minimize risk of adverse outcomes for research participants. | 1 | 2 | 1 | 9 | - | - | 1 | 2 | 8 | 2 |
| ...report the results of research to the scientific community. | 2 | 1 | 2 | 8 | - | - | **-** | 4 | 7 | 2 |
| I feel confident that I can identify the appropriate way to honor my ethical obligations: |  |  |  |  |  |  |  |  |  |  |
| ...when designing studies from which my patients/community members may benefit. | 1 | 2 | 4 | 6 | - | - | 1 | 2 | 9 | 1 |
| ...when designing studies in which my patients/community members may participate. | 1 | 2 | 4 | 5 | - | - | 1 | 2 | 8 | 2 |
| ...when obtaining my patient's/community member's informed consent to research. | 2 | 0 | 4 | 6 | 1 | - | **-** | 2 | 9 | 2 |
| ...when protecting my patients/community members from adverse outcomes during research. | - | 4 | 4 | 5 | - | - | - | 2 | 9 | 2 |
| ...when reporting back the results of research to my patients/community members. | - | 5 | 3 | 5 | - | **-** | **-** | 2 | 9 | 2 |
| ...when reporting the results of research to the scientific community | - | 1 | 3 | 9 | - | - | - | 3 | 8 | 2 |
| **Moral Reasoning** | **7%** | **17%** | **28%** | **46%** | **2%** | **-** | **3%** | **19%** | **61%** | **17%** |

**Table S3**: Distribution of Likert Scores by Item for Moral Motivation and Moral Action Constructs

|  | **Before the Course**  **(n)** | | | | | **After the Course**  **% (n)** | | | | |
| --- | --- | --- | --- | --- | --- | --- | --- | --- | --- | --- |
| **Item** | **Strongly Disagree** | **Disagree** | **Neutral** | **Agree** | **Strongly Agree** | **Strongly Disagree** | **Disagree** | **Neutral** | **Agree** | **Strongly Agree** |
| Protecting research subjects is primarily my responsibility. | - | - | - | 8 | 5 | - | - | 1 | 3 | 9 |
| I am responsible for balancing my role as a clinician/practitioner and as a researcher. | - | - | - | 7 | 6 | - | - | - | 7 | 6 |
| Principle investigators have an obligation to build trust with patients and community members. | - | - | - | 3 | 10 | - | - | - | 3 | 10 |
| **Moral Motivation** | **-** | **-** | **-** | **46%** | **54%** | **-** | **-** | **3%** | **33%** | **64%** |
| Navigating ethical dilemmas in the context of my research is within my control. | - | 3 | 2 | 7 | 1 | - | 2 | 3 | 7 | 1 |
| I have the ability to overcome barriers to conducting my research ethically. | - | - | 7 | 5 | 1 | - | - | 3 | 6 | 4 |
| I can access resources to help me navigate ethical challenges during my research. | - | 3 | 6 | 4 | - | - | - | - | 7 | 6 |
| **Moral Action** | **-** | **15%** | **38%** | **41%** | **5%** | **-** | **5%** | **15%** | **51%** | **28%** |
